# Chaos in Form and Color Yields to Harmony

**DOI:** 10.3201/eid2310.AC2310

**Published:** 2017-10

**Authors:** Byron Breedlove

**Affiliations:** Centers for Disease Control and Prevention, Atlanta, Georgia, USA

**Keywords:** art science connection, emerging infectious diseases, art and medicine, about the cover, Chaos in form and color yields to harmony, Colorful ensemble (Entassement regle), Wassily Kandinsky, bacteria

**Figure:**
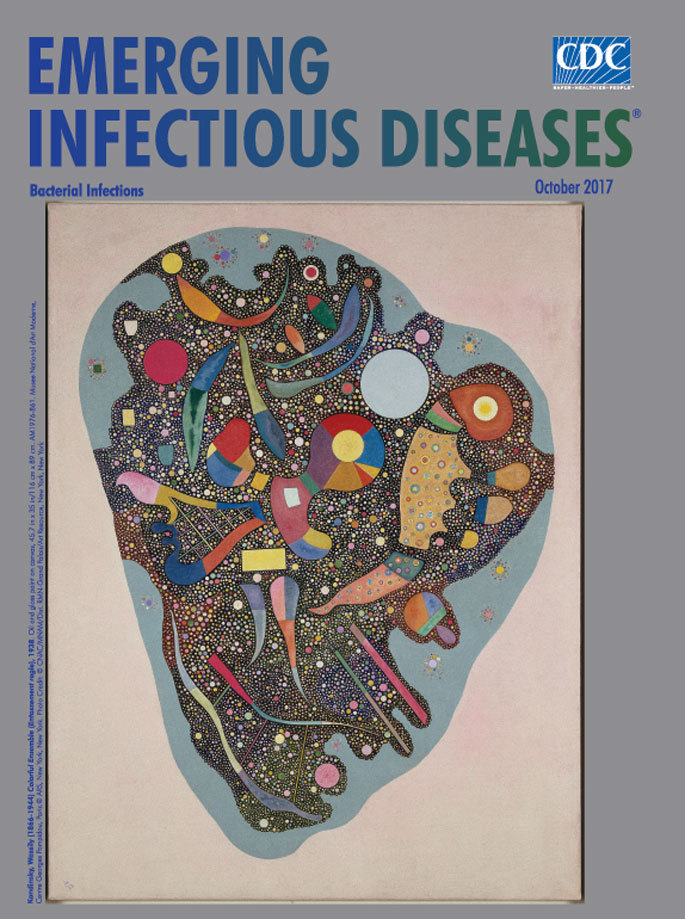
**Kandinsky, Wassily (1866–1944) Colorful Ensemble (Entassement regle), 1938. Oil and gloss paint on canvas, 45.7 in × 35 in/116 cm × 89 cm.** AM1976–861. Musee National d’Art Moderne, Centre Georges Pompidou, Paris. © Artists Rights Society (ARS), New York, New York. Photo Credit: © CNAC/MNAM/Dist. RMN-Grand Palais/Art Resource, New York, New York

Art critic Ossian Ward has written that Wassily Kandinsky strove “to evoke sound through sight and create the painterly equivalent of a symphony that would stimulate not just the eyes but the ears as well.” One of the great artists of the early 20th century, Kandinsky was born on December 4, 1866, in Moscow to well-educated, upper-class parents. From a young age, he exhibited interest in and sensitivity to sound and color. Although he attended drawing and music classes during his childhood, Kandinsky pursued law, ethnography, and economics when he enrolled the University of Moscow in 1886. A decade later he abandoned a career in teaching law to attend art school in Munich, Germany, where he met other like-minded young artists and co-founded “Phalanx,” an art school that spurned traditionalist approaches and conventions.

Ward notes that Kandinsky is believed to have had synesthesia and to have the experience of hearing color and seeing sound. Synesthesia is a mingling of sensory input in which stimulation in one sense, such as hearing, concurrently and consistently stimulates a sensation in another sense, e.g., vision or taste. Kandinsky often drew from the lexicon of music in naming or describing his works as compositions, impressions, and improvisations. Neurologist and author Richard E. Cytowic states that “Kandinsky was among the first to step off the path of representation that Western art had followed for 500 years, and his model for this new ‘symbolic’ form of art was music.”

For the last 11 years of his life, Kandinsky lived in Paris. During those years, according to his Centre Pompidou biography, “Kandinsky painted and drew prolifically, putting together an important body of work in which the common factor is the inspiration of images from biology, forms resembling embryos, larvae, or invertebrates, a minuscule population embodying the living.” This body of work, dubbed biomorphic abstraction, contrasts with the artist’s earlier abstract compositions that often featured straight lines, precise circles, and sharp angles.^1^ Art historian and painter Hajo Düchting observed that Kandinsky’s “basic geometrical forms dissolve into an unbelievable variety of shapes among which biomorphic ones predominate over those derived from geometrical shapes.”

A splendid example of Kandinsky’s biomorphic art, *Colorful Ensemble,* this month’s cover art, shows that regardless of this shift in approach, the artist’s lifelong passion for infusing his art with musical themes and imagery continued to be at the heart of his work. What at first viewing appears to be frenzied, haphazard chaos yields to a pervading sense of harmony. The neutral background issues a tranquil invitation to peer more carefully at the mass of teeming shapes and bold primary colors enclosed by the heart-shaped figure.

Kandinsky studs the blue border with small bejeweled images, miniature constellations of color and harmony that may be separating from the whole or moving toward absorption. Densely packed scores of perfect circles create a textured mosaic. Curious interspersed forms evoke musical imagery: the head of a guitar, a stringed harp, and breath marks. Other shapes within the painting resemble biologic structures, including flagella, ribosomes, and genetic material found in bacteria.

Kandinsky created his biomorphic art during a time of crucial discovery and innovation in treating bacterial infections. The physicist Ladislaus Laszlo Marton had recently examined biologic specimens with an electron microscope and published the first electron micrographs of bacteria. Alexander Fleming had discovered penicillin just a decade earlier, and antibiotics were for the first time being widely used to treat bacterial infections.

## Footnotes

1 According to the Tate Museum, biomorphic—derived from the Greek words “bios,” which means life, and “morphe,” which means form—was initially used during the 1930s “to describe the imagery in the more abstract types of surrealist painting and sculpture” (http://www.tate.org.uk/art/art-terms/b/biomorphic).
